# Obesity Promotes Cooperation of Cancer Stem-Like Cells and Macrophages to Enhance Mammary Tumor Angiogenesis

**DOI:** 10.3390/cancers12020502

**Published:** 2020-02-21

**Authors:** Lauren E. Hillers-Ziemer, Rachel Q. McMahon, Margaret Hietpas, Gretchen Paderta, Jennelle LeBeau, Jessica McCready, Lisa M. Arendt

**Affiliations:** 1 Program in Cellular and Molecular Biology, University of Wisconsin-Madison, 1525 Linden Drive, Madison, WI 53706, USA; lhillers@wisc.edu; 2 Department of Comparative Biosciences, School of Veterinary Medicine, University of Wisconsin-Madison, 2015 Linden Drive, Madison, WI 53706, USAmahietpas@wisc.edu (M.H.); paderta@wisc.edu (G.P.); 3 Department of Biological and Physical Sciences, Assumption College, 500 Salisbury Street, Worcester, MA 01609, USA; jennelle1296@live.com (J.L.);

**Keywords:** obesity, breast cancer, macrophages, cancer stem-like cells, angiogenesis

## Abstract

Obesity is correlated with worsened prognosis and treatment resistance in breast cancer. Macrophage-targeted therapies are currently in clinical trials, however, little is known about how obesity may impact treatment efficacy. Within breast adipose tissue, obesity leads to chronic, macrophage-driven inflammation, suggesting that obese breast cancer patients may benefit from these therapies. Using a high fat diet model of obesity, we orthotopically transplanted cancer cell lines into the mammary glands of obese and lean mice. We quantified changes in tumor invasiveness, angiogenesis and metastasis, and examined the efficacy of macrophage depletion to diminish tumor progression in obese and lean mice. Mammary tumors from obese mice grew significantly faster, were enriched for cancer stem-like cells (CSCs) and were more locally invasive and metastatic. Tumor cells isolated from obese mice demonstrated enhanced expression of stem cell-related pathways including *Sox2* and *Notch2*. Despite more rapid growth, mammary tumors from obese mice had reduced necrosis, higher blood vessel density, and greater macrophage recruitment. Depletion of macrophages in obese tumor-bearing mice resulted in increased tumor necrosis, reduced endothelial cells, and enhanced recruitment of CD8^+^ T cells compared to IgG-treated controls. Macrophages may be an important clinical target to improve treatment options for obese breast cancer patients.

## 1. Introduction

Almost 40% of the adult population in the United States is considered clinically obese [1]. Obesity, defined as a body mass index of greater than 30.0 kg/m^2^, is a known risk factor for the development of postmenopausal breast cancer [2,3]. Regardless of menopausal status, obese women diagnosed with breast cancer often have larger primary tumors, greater incidence of lymph node involvement, and poorly differentiated tumors compared to patients with a body mass index within normal range [2]. Obese patients have shorter disease-free survival rates [4,5] and a higher risk of breast cancer-related mortality than lean patients [6]. Further, obesity is associated with reduced efficacy of multiple breast cancer therapeutics [7,8,9]. Within breast tumors, cancer stem-like cells (CSCs), which comprise a minor population of tumor cells, are thought to underlie clinically aggressive tumor behavior such as local and distant tumor recurrence and increased resistance to conventional therapies [10,11]. Obesity may enhance CSCs within the breast tumors of obese patients, leading to the clinically observed increased incidence of both local and distant recurrence. 

Tumor associated macrophages (TAMs) have recently emerged as novel therapeutic targets in solid tumors. Within the tumor microenvironment, TAMs have been shown to establish niches within tumors conducive for CSC expansion [12,13,14], enhance angiogenesis and metastasis [15,16], and suppress tumor cell responses to different types of chemotherapy [17]. Multiple therapeutic strategies impacting TAM survival or activities have moved to phase I and phase II clinical trials [18,19]. While preclinical studies have shown promise for therapeutic efficacy, significant questions remain regarding identification of patients that are most likely to respond to these therapies. Obesity induces chronic, macrophage-driven inflammation within adipose tissue [20,21]. Within obese breast tissue, macrophages form crown-like structures surrounding necrotic adipocytes [22], leading to increased aromatase activity and secretion of inflammatory cytokines [23,24]. However, adipocytes have been implicated in resistance to angiogenesis-targeted therapies in obesity and may promote breast cancer growth through release of metabolic substrates and secretion of inflammatory cytokines [25,26,27]. A deeper understanding of how targeting macrophages may impact tumor growth in obese patients could improve stratification of patient cohorts for clinical trials.

Here, we sought to determine how obesity impacts the efficacy of therapeutic depletion of TAMs. We show that obesity, not consumption of a high fat diet, promoted mammary tumor growth, selected for CSCs, and enhanced metastasis. Tumors from obese mice were less necrotic and had a higher blood vessel density than tumors from lean mice. Using in vitro assays and isolation of primary TAMs, we observed cooperation between TAMs and obesity-induced CSCs to enhance angiogenesis. Further, we show that depletion of TAMs in vivo abrogated obesity-enhanced angiogenesis and reduced tumor growth. Overall, our findings suggest that obese breast cancer patients may have enhanced clinical benefit from macrophage-targeted therapies. 

## 2. Results

### 2.1. Effects of Obesity on Mammary Tumor Growth

**Figure 1:**
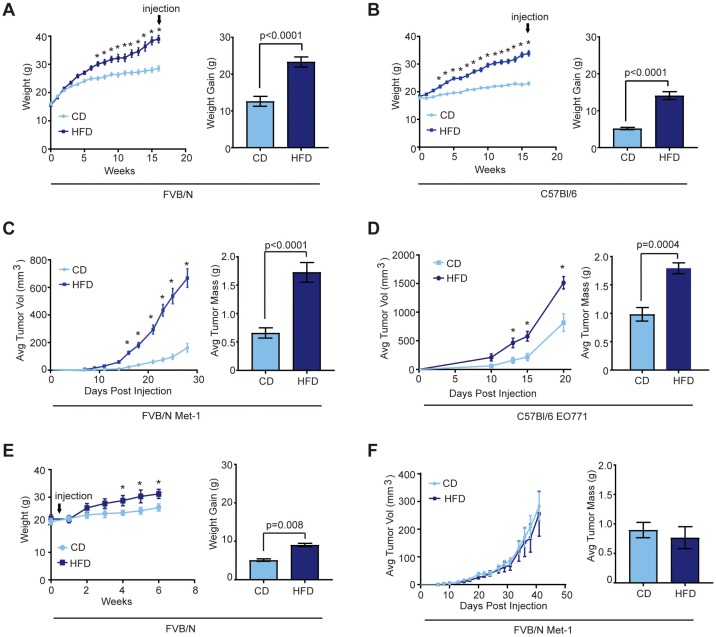
Obesity promotes tumor growth. Average body mass and weight gain of FVB/N (**A**, *n* = 12/group) or C57Bl/6 (**B**, *n* = 10/group) female mice fed a control diet (CD) or high fat diet (HFD) for 16 weeks prior to tumor cell transplant. Average body mass was analyzed using two-way ANOVA and Tukey’s multiple comparison test (* < 0.05); differences in weight gain were assessed with a *t*-test. Tumor growth curves and end stage tumor mass of Met-1 (**C**) or EO771 (**D**) tumor cells that were transplanted into the inguinal mammary glands of lean or obese FVB/N or C57Bl/6 female mice, respectively. Tumor growth was analyzed using two-way ANOVA and Tukey’s multiple comparison test (* < 0.05); differences in tumor weight assessed with *t*-test. (**E**) Average body mass and weight gain of female FVB/N mice placed on the CD or HFD at the time of Met-1 tumor cell transplant (*n* = 5/group). (**F**) Tumor growth curve and end stage tumor mass of Met-1 tumors that were transplanted into the mammary glands of FVB/N mice fed HFD or CD at the time of diet initiation. Tumor growth is represented as mean ± s.e.m.

To examine how obesity impacts mammary tumor formation, we utilized a high fat diet (HFD) model of obesity in mice. To identify strain-specific differences, FVB/N and C57Bl/6 female mice were randomized into groups and fed either control diet (CD) or HFD for 16 weeks to induce obesity. HFD-fed mice of both strains gained significantly more weight than those fed the CD (Figure 1A,B). We have previously observed that mice of both strains fed a HFD for 16 weeks have increased mammary gland weights and adipocyte size compared with CD-fed mice, as well as the formation of crown-like structures [28], consistent with changes observed in breast tissue of obese women [29]. After 16 weeks, Met-1 or EO771 mammary tumor cells were transplanted into the mammary glands of FVB/N or C57Bl/6 mice, respectively. Obese mice grew tumors that were significantly larger in volume and weight than those from lean mice (Figure 1C,D). Together, these results suggest that the rapid tumor growth observed in obese mice was not due to mouse strain or tumor cell line-specific effects. 

We have previously shown that 8-week-old FVB/N female mice are more weight gain resistant than those fed at 3-weeks of age [28]. When 8-week-old female FVB/N mice were transplanted with Met-1 cells then randomized into groups fed CD or HFD, HFD-fed mice demonstrated a mild, but significant weight gain, compared to CD-fed mice (Figure 1E). However, no significant differences were observed in volumes or weights of tumors that grew in the mammary glands of HFD or CD-fed mice (Figure 1F). These results suggest that obesity, rather than consumption of a HFD, enhanced mammary tumor growth. 

### 2.2. Obesity Promoted CSCs within Mammary Tumors

**Figure 2:**
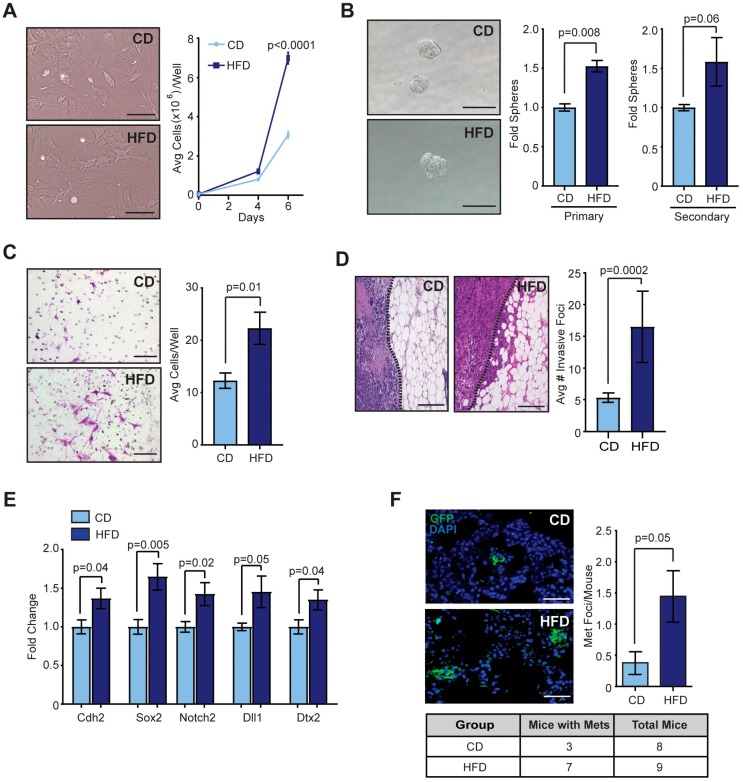
Mammary tumors from obese mice have increased properties of cancer stem-like cells. (**A**) Met-1 tumor cells isolated from lean and obese mice were plated in culture, and cell numbers were quantified after 4 and 6 days in culture (*n* = 3/group; two-way ANOVA with Tukey’s multiple comparisons test). (**B**) Isolated Met-1 tumor cells were plated in limiting dilution on non-adherent plates to form tumorspheres (*n* = 6/group; *t*-test). Tumorspheres were dissociated and re-plated to form secondary tumorspheres (*n* = 6/group; *t*-test). (**C**) Isolated Met-1 tumor cells were plated on collagen-coated migration chambers (*n* = 3/group; t-test). (**D**) Quantification of Met-1 tumor cell invasive foci into surrounding adipose tissue (*n* = 6/group; *t*-test). (**E**) Expression of markers of cancer stem-like cells (CSC)-like cells from isolated Met-1 tumor cells (*n* = 6/group; *t*-test). Expression differences were normalized to ribosomal protein lateral stalk subunit P0 (*Rplp0*) and represented as a fold change from controls. (**F**) Green fluorescent protein (GFP)-expressing Met-1 tumor cells were detected using immunofluorescence within the lungs (*n* = 8–9/group; *t*-test). Bars represent mean ± s.e.m. Magnification bars = 50 µm.

Since the tumors from obese mice demonstrated increased growth rates compared to those from lean mice, we dissociated Met-1 tumors from obese and lean mice, isolated single cells, and plated the cells in culture to quantify tumor cell proliferation. After 6 days in culture, we observed significantly increased numbers of Met-1 cells isolated from obese mice compared to those from lean mice (*p* < 0.0001, Figure 2A), suggesting that the tumor cells from obese mice had increased proliferation rates. The increased proliferation rate of tumor cells isolated from obese mice was maintained for at least three cell passages.

Given the increase in breast cancer recurrence and treatment resistance observed clinically in obese women [6,7,8,9], we hypothesized that obesity may increase CSCs within tumors. To test self-renewal, isolated Met-1 and EO771 tumor cells were plated in limiting dilution on non-adherent plates to form tumorspheres, then dissociated and re-plated to form secondary tumorspheres. Tumor cells from obese mice formed significantly more primary tumorspheres and increased secondary tumorspheres compared to those from lean mice (*p* = 0.008, Figure 2B and Figure S1A), suggesting that tumors from obese mice had an increased population of cells with ability for self-renewal. 

Cells within the CSC-enriched cell population have been shown to exhibit aspects of epithelial-to-mesenchymal transition, including decreased cell-to-cell attachments and acquisition of mesenchymal traits [30]. When isolated Met-1 or EO771 tumor cells were plated on collagen-coated migration chambers, we observed that tumor cells from obese mice were significantly more invasive than those from lean mice (*p* = 0.01, Figure 2C and Figure S1B). Consistent with increased invasive behavior in vitro, tumors from obese mice demonstrated significantly increased numbers of foci of tumor cell invasion into the surrounding adipose tissue compared to those from lean mice (*p* = 0.0002, Figure 2D and Figure S1C). Isolated Met-1 tumor cells from obese mice demonstrated increased expression of epithelial-to-mesenchymal transition marker N-cadherin (*Cdh2)* compared to those from lean mice, consistent with increased invasive behavior (*p* = 0.04, Figure 2E). In addition, Met-1 tumor cells from obese mice expressed significantly higher expression of CSC-associated genes *Sox2* and Notch Receptor 2 (*Notch2*), as well as Notch ligand *Dll1* and downstream regulator *Dtx2* (Figure 2E). Together, these results suggest that obesity promotes the expansion of CSCs within mammary tumors. 

Both CSCs and the epithelial-to-mesenchymal transition program have been implicated in the generation of distant metastases [30]. To examine metastasis, we identified micrometastases within tissue sections of lungs. In both FVB/N and C57Bl/6 mice, obese tumor-bearing mice had significantly greater numbers of metastatic lesions within the lungs than lean tumor-bearing mice (*p* = 0.05, Figure 2F and Figure S1D). These results suggest obesity promotes distant metastases from mammary tumors, possibly through expansion of CSCs. 

### 2.3. Obesity Enhances Angiogenesis in the Tumor Microenvironment

**Figure 3:**
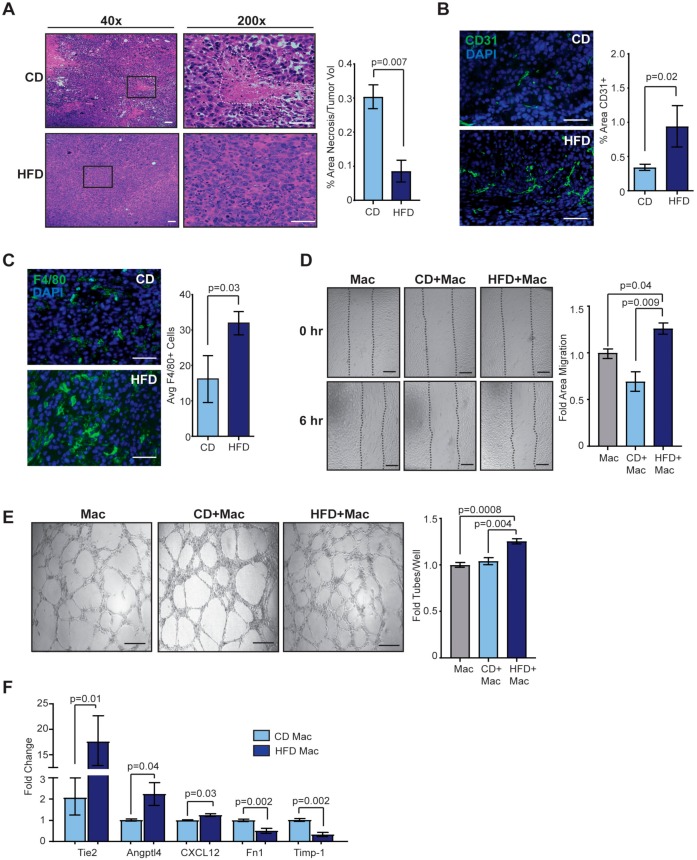
Tumor associated macrophages (TAMs) interact with tumor cells from obese mice to promote angiogenesis. (**A**) Necrotic area was quantified from Met-1 tumors isolated from lean or obese mice (*n* = 10/group, *t*-test). CD31 expression (**B**) and F4/80^+^ TAMs (**C**) were detected using immunofluorescence and quantified in Met-1 tumors (*n* = 6/group, *t*-test). Isolated Met-1 tumor cells were co-cultured with THP-1 differentiated macrophages and conditioned media was collected. Human umbilical vein endothelial cells were plated in wound healing assays (**D**) and networking assays (**E**) in the presence of conditioned media (*n* = 5/group, one-way ANOVA analysis with Tukey’s multiple comparison test). (**F**) Primary TAMs were isolated from Met-1 tumors. Expression differences were normalized to cyclophilin and represented as a fold change from controls (*n* = 6/group, *t*-test). Bars represent mean ± s.e.m. Magnification bars = 50 µm.

To investigate how obesity impacts the mammary tumor microenvironment to support rapidly growing tumors, we examined tissue sections from Met-1 and EO771 tumors from lean and obese mice. Although larger, Met-1 tumors from obese mice demonstrated significantly decreased necrosis compared to tumors from lean mice (*p* = 0.007, Figure 3A). An underlying cause of necrosis may be an insufficient blood supply [31]. To detect endothelial cells, tumors sections from Met-1 and EO771 tumors from obese and lean mice were stained with anti-CD31 antibodies. Met-1 and EO771 tumors from obese mice demonstrated significantly greater numbers of CD31^+^ cells within tumors than those from lean mice (*p* = 0.02, Figure 3B and Figure S2A). Together these results suggest that obesity promotes angiogenesis within the mammary tumor microenvironment. 

Within adipose tissue, obesity leads to increased number and size of adipocytes as well as enhanced macrophage recruitment. We hypothesized that these changes within mammary adipose tissue may also be present within the tumor microenvironment. We quantified adipocytes within Met-1 and EO771 tumors of lean and obese mice and observed no significant differences in either size or number of adipocytes (Figure S2B,C). Consistent with elevated macrophages within obese adipose tissue, significantly greater numbers of F4/80^+^ TAMs were observed in Met-1 and EO771 tumors from obese mice compared to lean mice (*p* = 0.03, Figure 3C and Figure S2D). These results suggest that obesity-induced inflammation within adipose tissue also results in enhanced TAM numbers within mammary tumors. 

Since significantly more TAMs were observed in tumors from obese mice, and macrophages have been shown to promote angiogenesis in obese mammary tissue [32], we hypothesized that TAMs may increase tumor angiogenesis. We co-cultured Met-1 tumor cells isolated from obese and lean mice with THP-1 differentiated macrophages and collected conditioned media. Conditioned media from co-cultured macrophages and tumor cells from obese mice significantly enhanced human umbilical vein endothelial cell migration in wound healing assays (Figure 3D) as well as network formation when plated on Matrigel (Figure 3E) compared to conditioned media from co-cultured macrophages and tumor cells from lean mice or conditioned media from macrophages alone. In contrast, conditioned media isolated from tumor cells or adipose-derived stromal cells from obese mice did not significantly enhance either endothelial cell migration (Figure S3A,B) or network formation (Figure S3C,D). These results suggest that TAMs and tumor cells from obese mice might cooperatively promote angiogenesis within the tumor microenvironment. 

**Figure 4:**
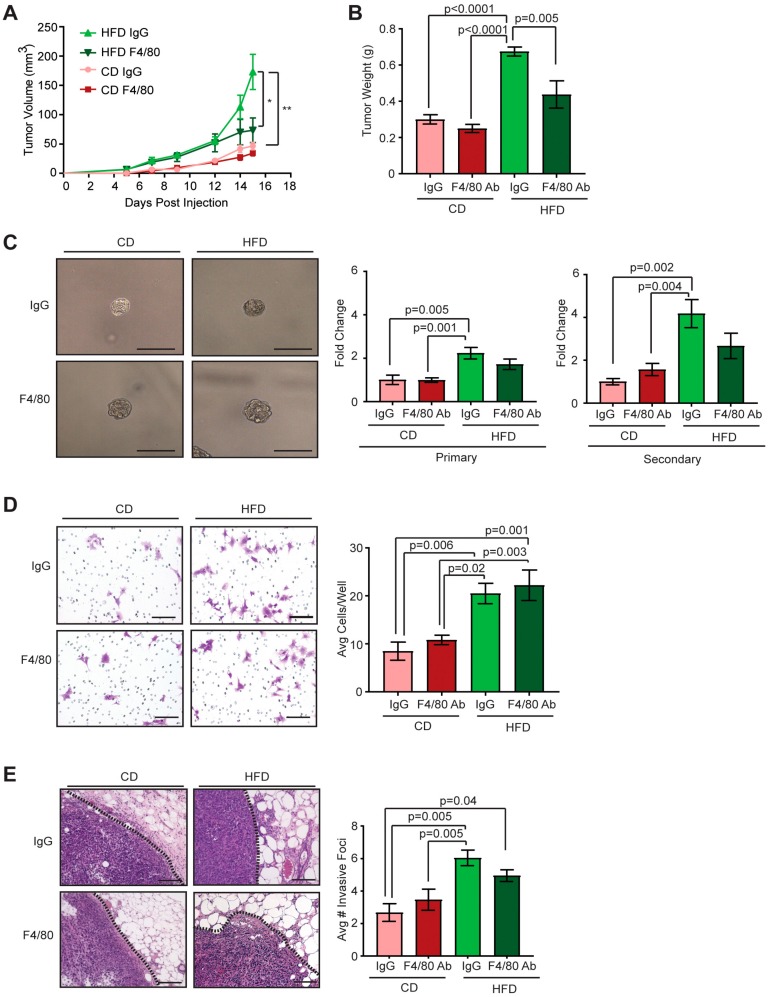
Depletion of macrophages has limited effects on functional CSCs in Met-1 tumors from lean or obese mice. Lean or obese FVB/N female mice were transplanted with Met-1 tumor cells, and received treatment with either anti-F4/80 or IgG control antibodies (*n* = 3–4/group). Tumor growth curves (**A**) and end stage tumor masses (**B**) from treated mice. Tumor growth and end stage tumor masses analyzed with two-way ANOVA with Tukey’s multiple comparison test (*p < 0.0001 HFD IgG compared to HFD F4/80; **p < 0.0001 HFD IgG compared to CD IgG and HFD IgG compared to CD F4/80). (**C**) Isolated Met-1 tumor cells were plated in limiting dilution on non-adherent plates to form tumorspheres (*n* = 3–4/group). Tumorspheres were dissociated and re-plated to form secondary tumorspheres (two-way ANOVA with Tukey’s multiple comparison test). (**D**) Isolated Met-1 tumor cells were plated on collagen-coated migration chambers, and invading cells were quantified (*n*= 3/group; two-way ANOVA with Tukey’s multiple comparison test). (**E**) Quantification of Met-1 tumor cell invasive foci into surrounding adipose tissue (*n* = 3–4/group; two-way ANOVA with Tukey’s multiple comparison test). Bars represent mean ± s.e.m. Magnification bars = 50 µm.

Macrophages demonstrate unique expression profiles in adipose tissue depending on extracellular cues [33], and we hypothesized TAMs isolated from tumors of obese mice may have altered expression of angiogenic factors compared to those from lean mice. To identify differences in expression of angiogenic factors, we isolated primary TAMs from Met-1 tumors from obese and lean mice and examined expression of known angiogenic factors. TAMs isolated from tumors from obese mice exhibited significantly increased expression of angiopoietin-like-4 (*Angptl4*) and *CXCL12*, as well as *Tie2* (Figure 4C). Tie2-expressing TAMs have been shown to promote angiogenesis in various tumor models [34]. Primary TAMs from tumors of obese mice also demonstrated significantly decreased expression of tissue inhibitor of metalloproteinase-1 (*Timp-1*) and fibronectin-1 (*Fn1*), which have been shown to inhibit angiogenesis [35], compared to those from lean mice (Figure 3F). These data suggest macrophages within tumors of obese mice may secrete higher levels of factors that enhance angiogenesis and decreased expression of factors that inhibit angiogenesis, leading to enhanced blood vessel density within tumors. 

### 2.4. Depletion of Macrophages in Obese Mice Did Not Alter CSCs within Met-1 Tumors

To investigate the effects of TAM depletion on tumor growth under conditions of obesity, we randomized lean and obese mice into treatment groups receiving either anti-F4/80 antibodies to deplete macrophages or IgG control antibodies and transplanted Met-1 tumor cells into mammary fat pads. No significant differences in weight were observed in either lean or obese mice as a consequence of antibody treatment (Figure S4A). Within the obese group, tumor-bearing mice receiving anti-F4/80 antibodies had significantly smaller tumors than those treated with IgG control (Figure 4A,B). In contrast, lean tumor-bearing mice treated with anti-F4/80 or IgG antibodies showed no differences in tumor growth (Figure 4A,B). Tumors from lean mice were similar in size and weight to those from obese mice treated with the anti-F4/80 antibodies (Figure 4A,B). To assess macrophage-depletion, we quantified F4/80^+^ TAMs within tumors sections. Tumors from macrophage-depleted lean and obese mice had significantly decreased numbers of F4/80^+^ TAMs within tumors than those treated with IgG control antibodies (Figure S4B). Together, these data suggest that macrophages enhance tumor growth under conditions of obesity. 

TAMs have been shown to enhance CSCs within the tumor microenvironment [12,14]. To determine the impact of macrophage depletion on CSCs, we plated dissociated single Met-1 tumor cells from obese and lean mice treated with either anti-F4/80 or IgG antibodies on non-adherent plates to form tumorspheres. Similar to untreated mice (Figure 2B), Met-1 cells isolated from IgG-treated obese mice demonstrated significantly increased formation of both primary and secondary tumorspheres compared to those from IgG-treated lean mice (Figure 4C). However, within each diet group, macrophage depletion did not significantly alter the ability of isolated tumor cells to form tumorspheres compared to tumor cells isolated from respective controls (Figure 4C). To assess changes in invasive behavior, isolated tumor cells were plated on collagen-coated migration chambers. Tumor cells isolated from obese mice treated with IgG antibodies were significantly more invasive compared to those from control lean mice (*p* = 0.006, Figure 4D). Macrophage depletion did not significantly alter tumor cell invasion compared to controls (Figure 4D). In vivo, tumors from IgG-treated obese mice demonstrated significantly increased numbers of invasive foci into the surrounding adipose tissue compared to those from IgG-treated lean mice (*p* = 0.005, Figure 4E). Consistent with in vitro invasion, macrophage depletion did not alter the number of invasive foci detected compared to controls (Figure 4E). These results suggest that depletion of TAMs does not significantly impact CSC numbers within Met-1 mammary tumors. 

### 2.5. Macrophage Depletion Reduces Angiogenesis in Tumors of Obese Mice 

**Figure 5:**
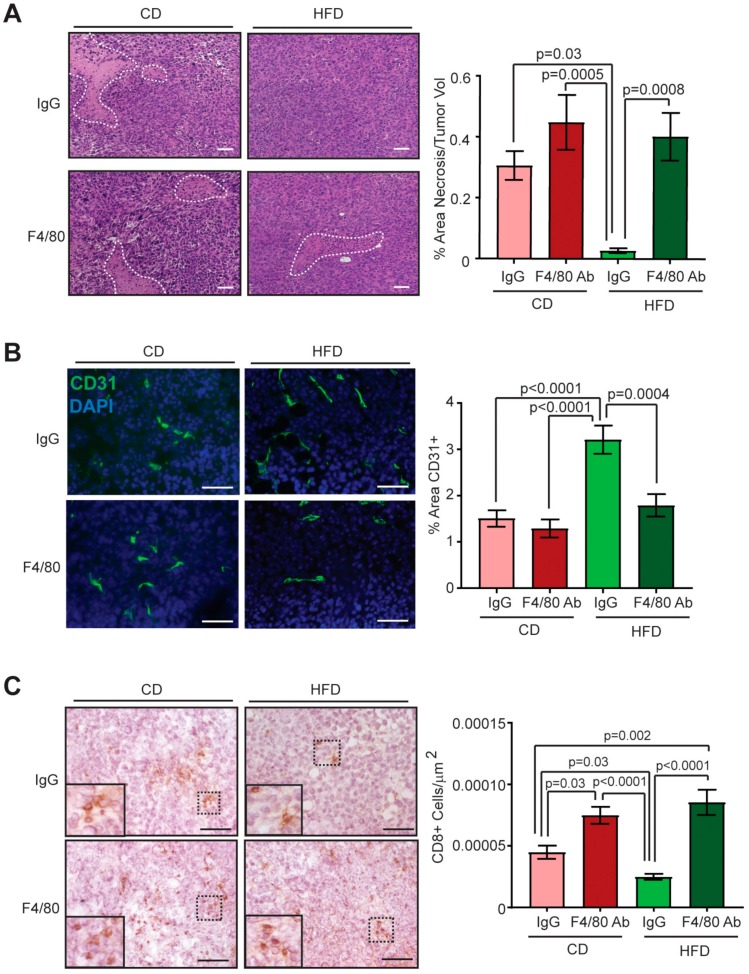
Depletion of macrophages in obese mice abrogates obesity-induced angiogenesis. (**A**) Percent necrotic area was quantified from tumor sections (*n* = 3–4/group; two-way ANOVA with Tukey’s multiple comparison test). An interaction between the variables of obesity and macrophage-depletion was observed (*p* = 0.06). (**B**) CD31 expression was detected using immunofluorescence in tumors from each group (*n* = 3–4/group; two-way ANOVA with Tukey’s multiple comparison test). A significant interaction between the variables of obesity and macrophage-depletion was observed (*p* = 0.02). (**C**) CD8^+^ T cells were quantified from tumors using immunohistochemistry (*n* = 3–4/group; two-way ANOVA with Tukey’s multiple comparison test). A significant interaction between the variables of obesity and macrophage-depletion was observed (*p* = 0.007). Bars represent mean ± s.e.m. Magnification bars = 50 µm.

To elucidate how depletion of TAMs impacted angiogenesis, we quantified necrosis in Met-1 tumors from lean and obese mice treated with anti-F4/80 or IgG antibodies. Consistent with untreated mice (Figure 3A), tumors from IgG-treated obese mice demonstrated significantly reduced necrosis compared to those from IgG-treated lean mice (*p* = 0.03, Figure 5A). Tumors from macrophage-depleted obese mice demonstrated significantly increased necrosis compared to those from IgG-treated obese mice (*p* = 0.0008, Figure 5A). No significant differences in necrosis were observed among tumors from lean mice treated with either anti-F4/80 or IgG antibodies (Figure 5A). To assess endothelial cells, tumor sections were stained with CD31 antibodies. Tumors from IgG-treated obese mice demonstrated significantly greater CD31^+^ endothelial cells compared to those from IgG-treated lean mice (*p* < 0.0001, Figure 5B). Tumors from macrophage-depleted obese mice demonstrated significantly reduced CD31^+^ endothelial cells compared to those from IgG-treated obese mice (*p* = 0.0004, Figure 5B). No significant differences in CD31^+^ endothelial cells were observed among tumors from lean mice treated with either anti-F4/80 or IgG antibodies (Figure 5B). A significant interaction between the variables of obesity and macrophage-depletion on the effects of CD31^+^ endothelial cells within tumors was observed (*p* = 0.02). Together, these data suggest that TAMs significantly enhance tumor angiogenesis under conditions of obesity. 

TAMs have been shown to be immunosuppressive in the tumor microenvironment [13]. To examine the effect of macrophage depletion on T cell recruitment, we stained tumor sections for CD8. Tumors from IgG-treated obese mice demonstrated significantly reduced CD8^+^ T cells compared to those from IgG-treated lean mice (*p* = 0.03, Figure 5C). Tumors from macrophage-depleted obese mice demonstrated significantly increased CD8^+^ T cells compared to those from IgG-treated obese mice (*p* < 0.0001, Figure 5C). Tumors from macrophage-depleted lean mice demonstrated significantly increased CD8^+^ T cells compared to those from IgG-treated lean mice (*p* = 0.03, Figure 5C). A significant interaction between the variables of obesity and macrophage-depletion on the effects of CD8^+^ T cells within tumors was detected (*p* = 0.007). Together these results suggest that depletion of TAMs under conditions of obesity significantly enhances T cell recruitment within mammary tumors. 

## 3. Discussion

Given the growing incidence of obesity, the number of breast cancer patients with the co-morbidity of obesity will likely continue to rise. Since obese patients have an increased risk for breast cancer treatment resistance and mortality than lean patients [6,7,8,9], identification of new treatment options could significantly benefit this patient population. Macrophage-targeted therapies are currently in clinical trials, however, little is known about how obesity impacts response to these therapies. Here, we show that macrophage depletion significantly reduces the growth of mammary tumors in obese mice, leading to increased tumor necrosis and decreased angiogenesis. Further, we observed that targeted depletion of macrophages led to enhanced recruitment of CD8^+^ T cells into the tumors of obese mice. Together, these results suggest that obese patients may strongly benefit from targeted-macrophage therapies in combination with chemotherapy or prior to immunotherapy. 

While TAM depletion reduced tumor growth in obese mice, we did not observe a significant impact on CSCs within the tumors of obese mice. These results were surprising, since TAMs have been shown to establish niches within the tumor conducive for CSC expansion [12,13,14]. Under culture conditions modeling obesity, metabolically activated macrophages also promoted CSC expansion in triple negative breast cancer cell lines [36]. While treatment of mice with anti-F4/80 antibodies resulted in reduced macrophage numbers, we did not observe complete depletion of TAMs. It is possible that limited treatment length and dose obscured the effect of macrophage depletion on CSCs. However, in addition to macrophage recruitment, obesity also leads to metabolic and hormonal abnormalities which may increase CSCs within the tumor microenvironment. We observed significant increases in *Sox2* and *Notch2* expression in tumor cells isolated from obese mice compared to those from lean mice. Sox2 has been shown to increase CSCs, enhance tumor cell invasion, and promote tamoxifen resistance in breast cancer lines [37,38]. Leptin, which is secreted by adipocytes and significantly enhanced in obesity, has been shown to promote CSCs [39], potentially through regulation of Notch signaling [40]. Adipocytes may also induce invasive and aggressive tumor cell behavior through secretion of cytokines [25], and we have previously observed that adipose-derived stromal cells isolated from the mammary glands of obese mice enhanced local invasion of Met-1 and EO771 cells through secretion of insulin-like growth factor-1 [41]. Interestingly, isolated tumor cells from obese mice retained the ability to proliferate at a higher rate through multiple passages, which may suggest that obesity induces epigenetic changes within breast tumors. 

Although promising in preclinical studies, anti-angiogenic therapies for cancer patients have not been largely successful. A recent study suggests that obesity promotes resistance to anti-vascular endothelial growth factor therapy through production of alternative angiogenic factors [27]. We observed significantly increased Tie-2 expression in macrophages isolated from tumors of obese mice, which may suggest that obesity enriches for a subpopulation of macrophages that are known to promote angiogenesis [42]. TAMs isolated from tumors from obese mice also secreted multiple factors that modify angiogenesis. Given the number of angiogenic factors that are produced by the macrophages under conditions of obesity, therapeutically targeting macrophages rather than individual angiogenic factors may lead to improved anti-angiogenic responses. Consistent with this idea, depletion of macrophages in obese mice resulted in significant decreases in endothelial cells and enhanced tumor necrosis. Within adipose tissue, adipocytes have also been shown to regulate angiogenesis through the secretion of adipokines [43], and adipokine secretion, including leptin, has been implicated in tumor angiogenesis in the context of obesity [44,45]. Although we observed increased tumor cell invasion into surrounding obese adipose tissue, we did not observe elevated numbers of adipocytes within the tumor mass. In contrast to adipocytes, TAMs comprise a significant fraction of the tumor stroma and are increased in tumors of obese mice. However, macrophages also express the receptor for leptin [46], and leptin secretion by adipocytes may enhance the angiogenic factors secreted by macrophages. We observed that depletion of TAMs resulted in reduced tumor growth, increased tumor necrosis, and diminished angiogenesis within tumors of obese mice. These results suggest that TAMs from tumors of obese mice significantly enhance angiogenesis and targeting the function of these cells may enhance anti-angiogenic responses. 

A challenge with clinical trials is the stratification of patient cohorts that are most likely to respond to treatment. Our data suggests that obese women may significantly benefit from macrophage-targeted treatment. A caveat to these studies is that we did not observe a significant decrease in CSCs within the tumors of obese mice in response to macrophage depletion. These results may suggest that additional therapeutic strategies, such as Notch inhibitors, which are also in clinical trials, may reduce treatment resistance in obese breast cancer patients [47]. Further work to understand how obesity enhances CSCs within tumors may identify additional therapeutic targets. As a consequence of macrophage depletion, we also observed an increase in CD8^+^ T cell recruitment, which was significantly enhanced in the tumors from obese mice. Macrophage-targeted therapies may also improve the efficacy of other immunotherapies in obese breast cancer patients. Understanding both the timing and the duration of macrophage-targeted therapies for use with other therapeutic agents will significantly enhance the utility of these therapies for obese women. 

## 4. Materials and Methods 

### 4.1. Animal Studies

All animal procedures were approved by the University of Wisconsin-Madison Institutional Animal Care and Use Committee (Animal Welfare Assurance Number (D16-00239).Female C57BL/6 (000664) and FVB/N (001800) mice were purchased from Jackson Laboratories and maintained according to the Guide for Care and Use of Laboratory Animals in AAALAC-accredited facilities. Eight-week-old female C57Bl/6 and three-week-old FVB/N mice were fed CD (10% kcal from fat, Test Diet; 58Y1) or HFD (60% kcal from fat, Test Diet; 58Y2) for 16 weeks to induce obesity. Purified diets contained equal amounts of vitamins and micronutrients. Body weights were measured weekly.

### 4.2. Cell Lines

THP-1 cells were obtained from American Type Culture Collection (ATCC; TIB202) and grown in RPMI with 10% FBS and 5% HEPES. Human umbilical vein endothelial cells (HUVEC; Lonza; cc-2519) were grown in endothelial cell growth media (EGM) bulletkit (Lonza; cc-5035). Adipose-derived stromal cells were isolated from the mammary glands of CD and HFD-fed mice as described [41]. EO771 cells were provided by Dr. Mikhail Kolonin [48] and were grown in RPMI (Corning; 10-040-CV) with 10% FBS. Met-1 cells were provided by Dr. Alexander Borowsky [49] and were transduced with lentivirus encoding green fluorescent protein (GFP). GFP^+^ cells were selected using fluorescence-activated cell sorting. Met-1 tumor cells and adipose-derived stromal cells were cultured in DMEM (Corning; 10-017-CV) with 10% FBS. All media contained 1% antibiotic/anti-mycotic solution (Mediatech; 30-004-CI), and cells were maintained at 37 °C in 5% CO_2_. All cultured cells were tested for mycoplasma prior to use in experiments (Idexx Bioresearch). 

### 4.3. Tumor Cell Line Transplantations

To generate tumors, 1 × 10^6^ EO771 or 5 × 10^5^ Met-1 cells were suspended in 2:1 Matrigel (Corning; 354234):DMEM and injected into the inguinal mammary glands of HFD or CD-fed C57Bl/6 or FVB/N female mice, respectively. Tumor diameters were measured using calipers three times weekly, and volume was calculated using 4/3πr^3^. End stage was defined when tumors reached the humane endpoint of 1.5 cm in diameter. A portion of each tumor was paraffin embedded or digested in DMEM:F12 (Corning; 10-090-CV) with 15 mg/mL collagenase I (Sigma; 1148089). Tumor organoids were cryopreserved and stored in liquid nitrogen vapor prior to use in experiments. Tumor organoids were resuspended in 10 mL cold PBS + 0.1% BSA. The mixture was passed through a 20G needle attached to a 10 mL syringe. The cells were centrifuged at 4 °C for 5 min at 233 × *g* and the supernatant was aspirated and discarded. Cells were resuspended in 5 mL 0.05% trypsin and incubated for 10 min at 37 °C. The cells were pelleted, then suspended in cell culture media supplemented with 100 μL of 5 mg/mL DNase, and passed through a 40 μM filter into a 50 mL conical tube. The cells were centrifuged for at 4 °C for 5 min at 233 × *g* and the supernatant was aspirated and discarded.

### 4.4. Cancer Stem-Like Cell Assays

Tumor organoids were dissociated to single cells as described [41]. To measure cell growth, 1 × 10^5^ Met-1 or EO771 tumor cells isolated from CD or HFD-fed mice were plated in triplicate on 35 mm^2^ tissue culture plates (Greiner; 627-160) in cell growth media. After 4 days, cells were trypsinized and counted using a hemocytometer, then re-plated on 100 mm^2^ tissue culture plates (Greiner; 664170). Primary tumor cells were incubated for an additional 2 days and counted. To assess invasion, 2.5 × 10^4^ Met-1 or 5 × 10^4^ EO771 primary tumor cells were plated in triplicate in serum-free cell growth media on rat tail collagen-I (Corning; 354236) coated 8.0 μm migration chambers (Corning; 353097) with three biological replicates. Invasion toward DMEM or RPMI supplemented with 10% FBS was measured after 48 h. Invasion inserts were formalin-fixed and stained with 0.1% crystal violet. Four images of each invasion insert were taken at 100× magnification on a Nikon Eclipse E600 Microscope with a QICAM Fast 1394 camera and quantified using ImageJ (NIH) with cell counter plug-in. To quantify tumorspheres, 1000 Met-1 or 500 EO771 primary tumor cells were plated in triplicate on 24-well ultra-low adherent plates (Costar; 3473) in cell growth media for 5 days. Tumorspheres were collected, spun down at 17 × *g* for 5 min and counted using a light microscope in a 96-well plate. Primary tumorspheres were trypsinized with 0.05% trypsin (Corning; 25-052-CI) for 10 min at 37 °C, spun down at 193 × *g* for 5 min, and plated on a 24-well ultra-low adherent plate for secondary tumorsphere formation. Secondary tumorspheres were incubated at 37 °C in 5% CO_2_ and counted after 5 days. Tumorsphere assays were plated in triplicate.

### 4.5. Histology, Immunohistochemistry, and Immunofluorescence

Paraffin-embedded tissues were sectioned and stained with Hematoxylin and Eosin (H&E) by the Experimental Pathology Laboratory (Carbone Cancer Center, University of Wisconsin-Madison). Tumor necrosis, adipocytes, and invasive tumor foci were identified using H&E slides. Necrosis was defined as homogeneous clusters and sheets of degenerating and dead cells. Percent necrosis was quantified by measuring the area of necrosis divided by the total tumor area of each image. To quantify tumor adipocytes, adipocytes within the tumor boundary were counted using ImageJ. Invasive foci were quantified as described [41]. Tissue staining for CD31 (Biolegend; clone 390; 102401), F4/80 (Biolegend; clone BM8; 123102), and CD8 (Thermo Fisher; clone 53-6.7; 14008185) was performed as described [41]. To quantify F4/80^+^ cells, images were divided into four quadrants and the number of positive and negative cells in the top right quadrant for each image was counted. The area of CD31^+^ staining was quantified using ImageJ. CD8^+^ cells were quantified and divided by the total tumor area on each image. Five images were taken for each tumor and quantified from six tumors/group. Blinded tissue sections were imaged using a Nikon Eclipse E600 Microscope and QICAM Fast 1394 camera.

### 4.6. Metastasis Quantification

Lung sections were stained for GFP (New England Bioscience; NB100-1678) for FVB/N mice and cytokeratin 5 (Biolegend; clone Poly19055; 905501) for C57Bl/6 mice. Blinded tissue sections were scanned for metastases and imaged. To quantify metastases, lesion count per lung and metastatic incidence per group was counted.

### 4.7. Endothelial Cell Assays

For macrophages, 5 × 10^6^ THP-1 cells were treated with 200 nM phorbol 12-myristate 13-acetate (PMA, Sigma Aldrich P1585) for 3 days. Following differentiation, macrophages were washed twice with PBS, and 2.5 × 10^6^ Met-1 tumor cells were cultured with THP-1 cells for 24 h. Equal numbers of adipose-derived stromal cells from non-tumor bearing CD and HFD-fed mice and Met-1 tumor cells isolated from CD and HFD-fed mice were grown on 100 mm^2^ tissue culture plates until confluency. For generation of conditioned media, cells were washed with PBS, then EGM media without supplements was added to the cells. After 24 h, conditioned media was collected, passed through 0.2 μm filters (Fisher Scientific; 09-720-004), aliquoted, and stored at -80 °C for use in experiments. For the wound healing assay, 5 × 10^4^ HUVEC cells/well were plated on 12-well plates for 24 h until confluent in duplicate. At t = 0, a scratch was made through the center of the well a p200 pipette tip, the cell growth media was replaced with conditioned media, and the scratch was imaged. The plate was incubated for 6 h and the scratch was re-imaged. Area of the scratches were calculated using NIS Elements Imaging Software, and difference in area between t = 0 and t = 6 was calculated. For network assays, 5 × 10^4^ HUVEC cells suspended in conditioned media were plated on growth factor-depleted Matrigel (Corning; 354483) on 96-well plates in triplicate. HUVEC were incubated for 6 h, then the wells were imaged, and the number of branches formed was quantified. Data are represented as a fold change of the control samples using three biological replicates.

### 4.8. Macrophage Isolation and Quantitative RT-PCR

Dissociated cells from primary tumors were incubated with anti-F4/80 antibodies (Biolegend; clone BM8; 123102) bound to sheep anti-rat IgG-conjugated beads (Life Technologies; 11035) and isolated according to manufacturer’s instructions. RNA was isolated from macrophages and tumor cells using TRIzol (Life Technologies; 15596026) and purified using Qiagen RNeasy Mini Kit (Qiagen; 74104). RNA was reverse transcribed using the High Capacity cDNA Reverse Transcription Kit (Applied Biosciences; 4368814) and Techne Thermal Cycler or Biometra Tone Thermal Cycle. Quantitative PCR was performed using iTaq SYBR Green Supermix (Bio-Rad; 172-5121) with a Bio-Rad CFX Connect Real-Time PCR Detection System (Bio-Rad). Transcripts were normalized to housekeeping genes cyclophilin or ribosomal protein lateral stalk subunit P0 (*Rplp0*), and data was analyzed using the ∆∆Cq method. Primer sequences are listed in Table S1.

### 4.9. Macrophage Depletion

CD and HFD-fed mice were randomized into treatment groups receiving InVivoMAbs rat IgG2b isotype control (BioXCell; clone LTF-2; BE0090) or anti-mouse F4/80 antibodies (BioXCell; clone C1:A3-1; BE0206). A loading dose of 400 ng/mouse of F4/80 or IgG antibodies were administered subcutaneously 24 h prior to mammary transplantation of 5 × 10^5^ Met-1 cells. Mice received antibody treatments of 200 ng/mouse every other day for 2 weeks following transplant [50].

### 4.10. Statistics

Results were expressed as mean ± s.e.m. unless stated. Statistical differences were determined using Student’s *t*-test or one-way ANOVA with Tukey’s multiple comparison test. For macrophage depletion experiments and tumor growth, differences were detected using two-way ANOVA analysis with Tukey’s multiple comparison test. *p*-values of 0.05 or less were considered significant. Statistical analyses were conducted using GraphPad Prism 8.3.1 (GraphPad Software, San Diego, CA, U.S.A.).

## 5. Conclusions

Obesity is significantly correlated with a worsened prognosis in breast cancer patients and decreased response to multiple cancer therapies. Given the growing incidence of obesity, the number of obese women diagnosed with breast cancer will likely continue to rise. Macrophage-targeted therapeutics are currently in clinical trials, however, little is known about how the co-morbid condition of obesity may impact treatment efficacy. In this work, we observed that obesity promoted the growth of invasive, metastatic mammary tumors with increased numbers of tumor associated macrophages. Depletion of macrophages in obese mice led to reduced tumor growth, decreased angiogenesis within tumors, and increased recruitment of CD8^+^ T cells compared to controls. However, depletion of macrophages did not significantly reduce CSCs within the mammary tumors, which may suggest that additional targeted therapies are necessary to reduce tumor recurrence or metastasis. Understanding the impact of targeting macrophages on tumor growth under conditions of obesity may help to stratify patients in clinical trials and identify patients that will most benefit from these therapeutic options.

## Supplementary Materials

The following are available online at https://www.mdpi.com/2072-6694/12/2/502/s1, Figure S1: EO771 tumors from obese mice have increased properties of CSCs, Figure S2: EO771 tumors from obese mice demonstrate enhanced angiogenesis and recruitment of tumor-associated macrophages, Figure S3: Met-1 tumor cells and adipose-derived stromal cells isolated from obese mice do not enhance endothelial cell migration, Figure S4: Treatment with anti-F4/80 antibodies reduces TAMs, Table S1: Primers used for quantitative RT-PCR analysis.
